# A Lung Cancer Patient Harboring a Rare Oncogenic EGFR Exon 20 V786M Mutation Responded to a Third-Generation Tyrosine Kinase Inhibitor: Case Report and Review of the Literature

**DOI:** 10.3389/fonc.2022.912426

**Published:** 2022-05-18

**Authors:** Qi Zhu, Mingyun Jiang, Wenfei Li, Shuangli Sun, Jisheng Li, Justin Stebbing, Xiaodong Liang, Ling Peng

**Affiliations:** ^1^Cancer Center, Department of Pulmonary and Critical Care Medicine, Zhejiang Provincial People’s Hospital, Affiliated People’s Hospital, Hangzhou Medical College, Hangzhou, China; ^2^Graduate Department, Bengbu Medical College, Bengbu, China; ^3^Department of Biochemistry & Molecular Biology, Shandong University, Jinan, China; ^4^Department of Medical Oncology, Qilu Hospital, Cheeloo College of Medicine, Shandong University, Jinan, China; ^5^Division of Cancer, Department of Surgery and Cancer, Imperial College London, London, United Kingdom; ^6^Cancer Center, Department of Radiation Oncology, Zhejiang Provincial People’s Hospital, Affiliated People’s Hospital, Hangzhou Medical College, Hangzhou, China

**Keywords:** NSCLC, EGFR, rare mutation, tyrosine kinase inhibitor, immune checkpoint inhibitor

## Abstract

**Background:**

Epidermal growth factor receptor (EGFR) tyrosine kinase inhibitors (TKIs) are effective treatments for non-small cell lung cancer (NSCLC) patients with activating EGFR mutations. There are many uncommon and rare mutations in the EGFR gene. The efficacy of the EGFR-TKIs is largely unknown for cancers harboring uncommon or rare EGFR mutations.

**Case Presentation:**

A 69-year-old woman was diagnosed with adenocarcinoma cT4N2M1c, stage IVB. Next-generation sequencing (NGS) confirmed a rare EGFR V786M mutation. During chemotherapy, immune checkpoint inhibitor (ICI), and anti-angiogenic treatment, no radiological response was observed. Subsequent third-generation EGFR TKI showed a remarkable therapeutic effect. Structural prediction revealed that the V786M mutation induces conformational change at the dimer interface, without altering the ATP binding to the EGFR tyrosine kinase domain (TKD). Consistently, docking simulations indicated that the affinity of ATP to the V786M mutant was not disturbed, which explained the TKI sensitivity.

**Conclusions:**

Our data confirmed the activating role on EGFR V786M mutation. Together with structural predictions and clinical evidence for activity of TKIs against EGFR V786M mutations, these findings warrant further investigation.

## Background

Epidermal growth factor receptor (EGFR) mutations are the second frequent oncogenic driver event in non-small cell lung cancer (NSCLC) (1). Classical mutations such as exon 19 deletions and the L858R point mutation comprise the majority of EGFR mutations. However, low-frequency mutations occur within exons 18–25 of the EGFR gene in NSCLC including point mutations, deletions, insertions, and duplications, which are defined as uncommon mutations which account for 10%–20% of EGFR mutations (2).

Despite an increased application of more sensitive detection platforms to identify uncommon and rare EGFR mutations in NSCLC patients, our understanding of mutations is relatively poor compared to classical mutations. Clinical data related to these mutations are lacking due to their low incidence and difficulties in clinical trial recruitment, which results in a paucity of effective treatment strategies for uncommon or rare EGFR mutations.

Exon 20 insertions comprise 1%–10% of uncommon EGFR mutations (3). EGFR exon 20 insertions are associated with *de novo* resistance to EGFR TKIs and correlate with poor prognosis (4). A number of rare non-insertion point mutations in EGFR exon 20 have been also described in NSCLC (5).

Here we present a case of EGFR exon 20 V786M who was initially treated with chemotherapy plus immune checkpoint inhibitor and anti-angiogenic agents but did not yield satisfactory responses. However, gefitinib was associated with progression of intracranial metastases given as third-line therapy, while a third-generation TKI almonertinib yielded in a partial response both in lung and in intracranial metastases. Of note, we used I-TASSER to predict the 3D structure of EGFR V786M to investigate the oncogenic role of the V786M mutation and the efficacy of TKIs in the treatment of EGFR V786M mutant NSCLC patients (6).

## Case Presentation

**Figure 1:**
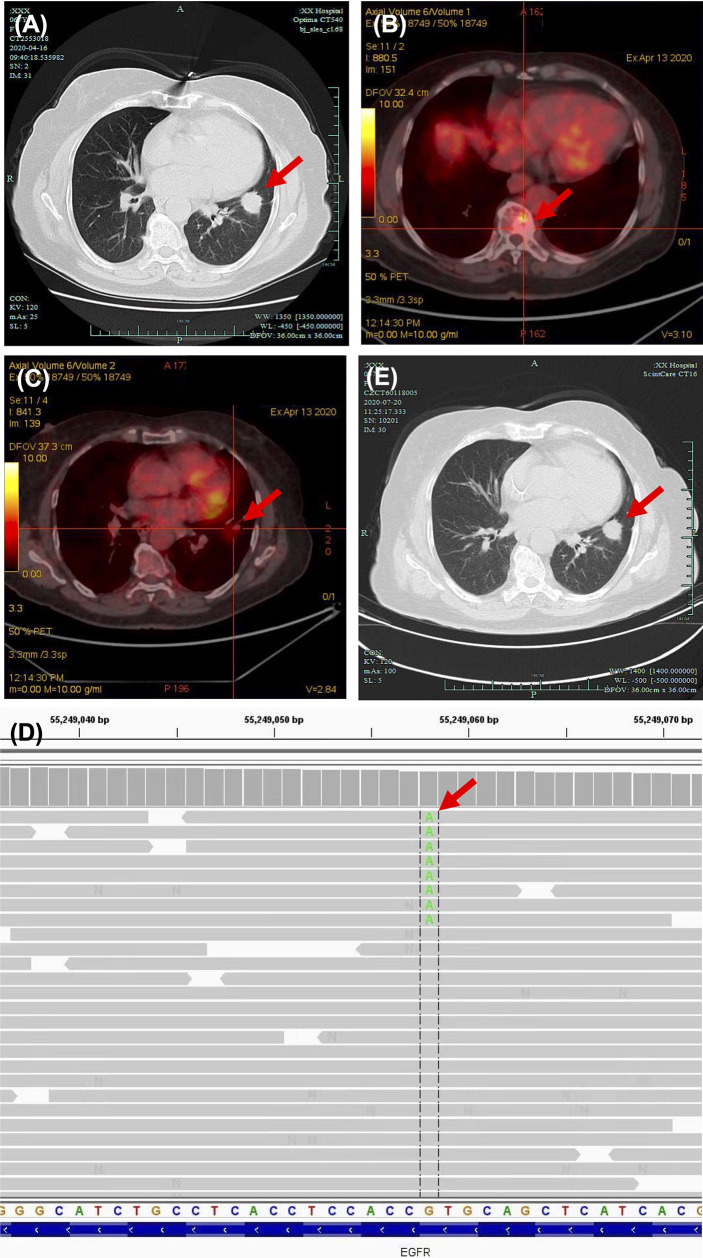
Diagnosis of EGFR V786M mutation and first-line therapy. **(A)** CT scan of lung window captured on April 16, 2020. Elevated SUV of bone lesion **(B)** and lung nodule **(C)** in PET-CT scan.**(D)** Next-generation sequencing (NGS) panel results showed an epidermal growth factor receptor (EGFR) mutation V786M in exon 20. **(E)** CT scan of the lung window captured on July 20, 2020, post first-line therapy. Significant abnormal findings noted (*arrow*).

A 69-year-old never-smoker woman in good clinical conditions was referred to a local hospital due to a solid lesion in the left lung on April 2020 (**Figure 1A**). Subsequent bronchoscopy revealed a lung adenocarcinoma, confirmed by TTF-1 and napsin A immunohistochemistry positivity. A brain MRI revealed no abnormalities Positron emission tomography (PET) CT scan indicated elevated standardized uptake value (SUV) of bone lesion (**Figure 1B**) and lung nodule (**Figure 1C**). The CT scan also revealed metastases in mediastinal lymph nodes and in the multiple vertebra; the final staging was cT4N2M1c (stage IVB). Next-generation sequencing (NGS) of tumor tissue revealed a variant (GUG > AUG) in the exon 20 of EGFR, resulting in amino acid substitution V786M (Val786Met) (**Figure 1D**).

The local doctors considered the patient non-eligible for targeted therapy due to lack of evidence. A first-line therapy of pemetrexed and carboplatin combined with anti-programmed death-1 (PD-1) antibody tislelizumab was used for 6 cycles, with best response of stable of disease (**Figure 1E**). No serious adverse events were observed. The patient went on a routine follow-up. In April 2021, progressive disease was defined due to the appearance of intracranial metastases (**Supplementary Figures S1A, B**). The primary lung cancer remained stable (**Supplementary Figure S1C**).

**Figure 2:**
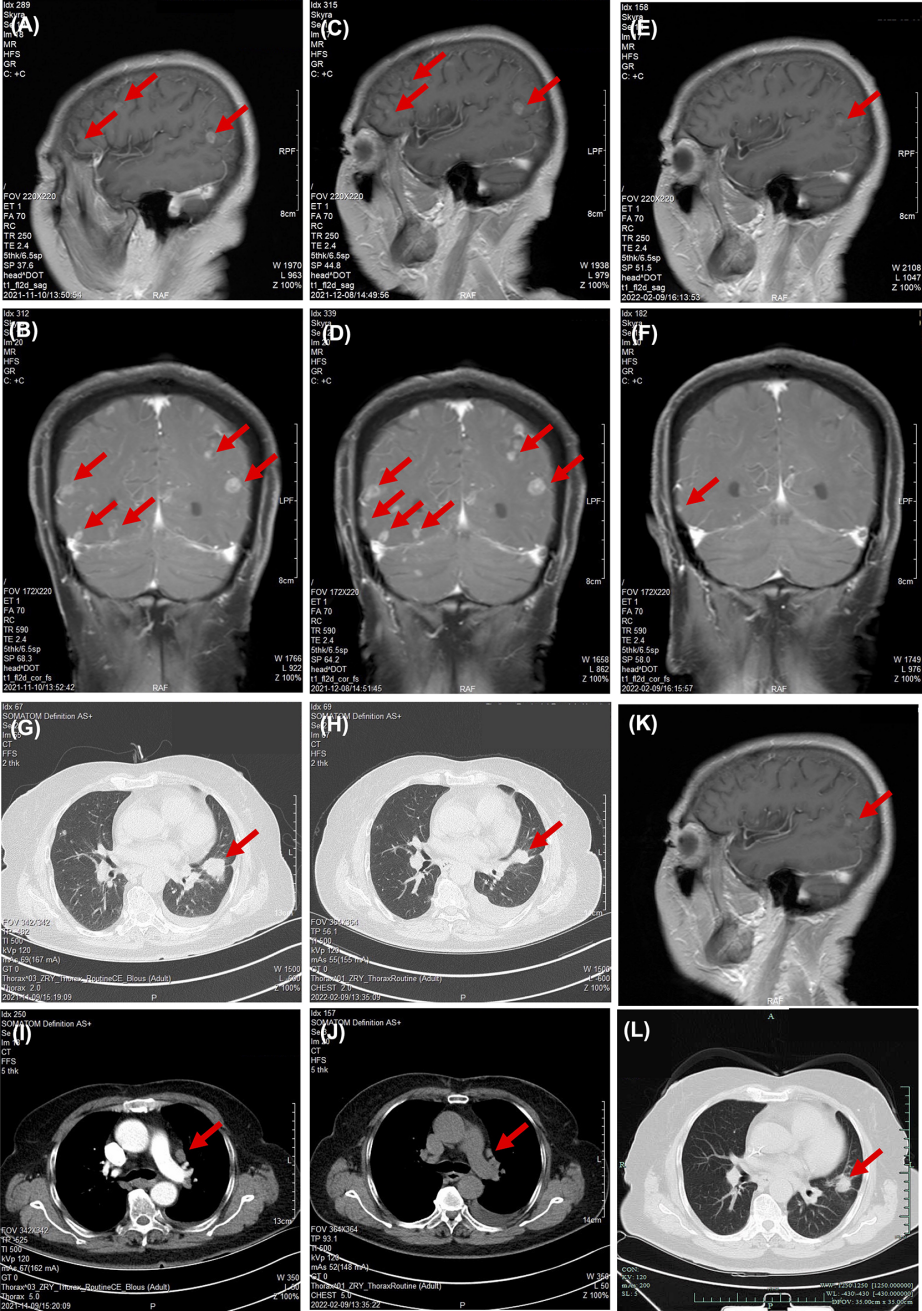
Responses of EGFR TKIs as third- and fourth-line therapy. Chest scanning CT before and after EGFR TKI treatment: **(A, B)** Brain MRI before gefitinib on November 10, 2021. **(C, D)** Brain MRI after gefitinib on December 8, 2021. **(E, F)** Brain MRI after almonertinib on February 9, 2022. **(G, H)** Chest CT scan before gefitinib on November 10, 2021. **(I, J)** Chest CT scan after almonertinib on February 9, 2022. **(K, L)** Chest CT and brain MRI 4 months post-almonertinib on April 14, 2022. Significant abnormal findings noted (*arrow*).

**Figure 3:**
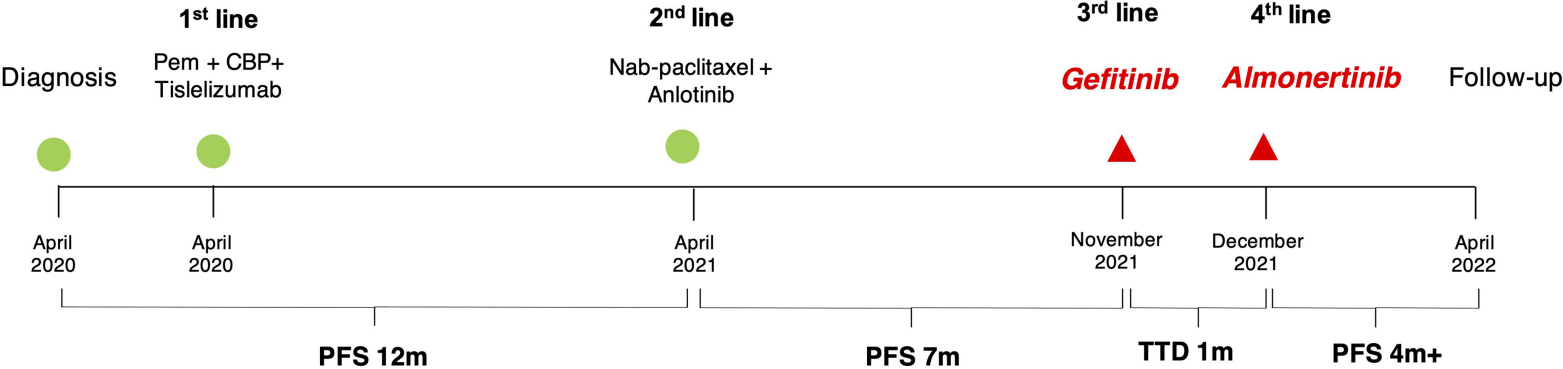
Timeline of patient history. TTD, time to discontinuation; PFS, progress-free survival; Pem, pemetrexed; CBP, carboplatin.

A second-line therapy of 4 cycles of nab-paclitaxel plus vascular endothelial growth factor receptor (VEGFR) TKI anlotinib was used, with best response of stable disease (**Supplementary Figures S1D–F**). Anlotinib was used for another 4 months for maintenance therapy (**Supplementary Figures S1G–I**). In November 2021, the patient complained of dizziness and fatigue. Brain MRI detected multiple intracranial metastases (**Figures 2A, B****)**. A multidisciplinary team discussion was held for this patient of medical oncologist, radiologist, thoracic surgeon, and pathologist. Despite the limited literature data available on the variant, we considered the patient eligible for treatment with gefitinib (250 mg/day), which started on November 10, 2021. A repeated MRI scan showing multiple brain lesions on December 8, 2021, suggested that new metastases were observed after 1 month of gefitinib (**Figures 2C, D****)**. A third-generation EGFR TKI almonertinib was used as fourth-line therapy. After 1 week of almonertinib treatment, the clinical conditions of fatigue and dizziness disappeared. A brain MRI and chest CT scan (**Figures 2E, F****)** and an indicated shrinkage of intracranial metastases and primary lung cancer (**Figures 2G–J**) were assessed as a partial response. A follow-up radiological examination of chest CT and brain MRI on April 14, 2022, confirmed the response as partial response (**Figures 2K, L**). The treatment timeline of this patient is illustrated in **Figure 3**.

**Figure 4:**
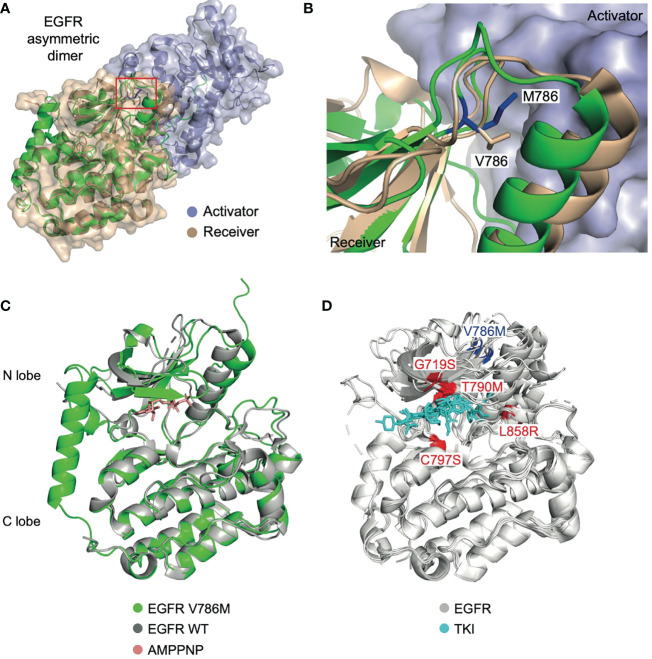
EGFR V786M mutation may facilitate the dimerization of EGFR, while it does not affect the binding of TKI. **(A)** Structure of the EGFR TK domain in the asymmetry dimer (PDB: 4LL0). The activator kinase is colored in slate, the receiver kinase in wheat. The predicted 3D structure of the V786M mutation is shown in cartoon (green) with V786 indicated in a red box. The modeled complex structure of the EGFR : EGFR homodimer, in which the N-lobe of the first EGFR (receiver) binds to the C-lobe of the second EGFR (activator) in a head-to-tail manner. **(B)** A close-up view of the dimer interface. M786 (blue) may strength the interaction between the EGFR dimer by protruding into the activator kinase domain (slate surface). **(C)** Structural alignment between EGFR V786M (green) and the wild-type protein (grey) with AMPPNP (red) bound (PDB: 3VJO). V786M induces conformational change in the N lobe region. **(D)** Analysis of EGFR mutations (PDB: 5ZWJ, 5GNK, 5GTY, 4ZAU, 4I22, 3UG2, 5Y9T, 6LUD). V786M locates in a different position from previously reported EGFR mutations which affects the binding of TKI (cyan) such as G719S, T790M, C797S, and L858R (highlighted in red). TK domain; tyrosine kinase domain. TKI, tyrosine kinase inhibitor; WT, wild-type.

To investigate the oncogenic role of the V786M mutation, we predicted the 3D structure of EGFR V768M mutation using publicly available tools (**Figure 4A**). V786 is located in the N-terminus of the receptor at the dimerization interface. While some frequently occurring point mutations such as G719S, T790M, and L858R have been well studied, the unexpected location of V786M led us to investigate its action in pathological state and the TKI efficacy. Structural analysis indicated that V786M protrudes into the kinase dimer interface and may increase the dimer interaction due to the larger size of methionine (**Figure 4B**). Intriguingly, the active state of EGFR is achieved through a “head-to-tail” configuration in asymmetric dimers and V786M could stabilize the interaction to keep the kinase domain for autophosphorylation and downstream signaling (**Figures 4C, D****)**. These results indicated that EGFR V786M mutation may facilitate the dimerization of EGFR, while it does not affect the binding of TKI.

## Discussion and Conclusions

Here, we report the case of a patient affected by advanced lung adenocarcinoma harboring a rare missense mutation in the exon 20 of the EGFR gene that resulted in an amino acid change (V786M). EGFR exon 20 V786M mutation was first reported in 2008 (7). The percentage of V786M mutation was 0.5% from 7,099 cancer patients in The Cancer Genome Atlas (TCGA) database (8).

The sensitivity of uncommon and rare mutations has been explored in cell line models. The evaluation of drug sensitivity of uncommon EGFR mutations using the mixed-all-nominated-mutants-in-one (MANO) method in Ba/F3 cells indicated that V786M might be sensitive to second- or third-generation TKIs than first-generation TKI (9). Owing to their molecular structures, second- or third-generation drugs have broader inhibitory profiles than the first-generation agents. In clinical patients, such rare mutations are generally sensitive to second- and third-generation TKIs than first-generation TKIs. Afatinib has been reported in 693 cases of NSCLC harboring uncommon EGFR mutations (10). Third-generation TKI osimertinib has also demonstrated potent activity against uncommon EGFR mutations in NSCLC (11). With the development of techniques, the clinical response of targeted drugs is also being tested in patient-derived organoids (PDOs) (12). The promise of PDOs as a patient-proximate culture system has led to great progress, with an increasing number of models emerging recently. However, the clinical application and guidance for PDOs are still facing many problems. Although both second- and third-generation TKIs have clinical activity against brain metastases in patients with EGFR mutation-positive NSCLC, data are lacking regarding their activity against brain metastases with EGFR uncommon mutations. A retrospective study involving 21 NSCLC patients of EGFR uncommon mutation with brain metastases indicated that EGFR-TKI treatment specifically to the brain metastases can prolong survival in these patients (13).

As many cancers have “subclonal” EGFR mutations with a low variant allele frequency, these subclonal mutations may exist in isolation or coexist with an independent common or uncommon EGFR mutation (14). Although TKIs are generally recommended in EGFR-mutant lung adenocarcinoma, little is known concerning the EGFR activation status related to rare mutations and their sensitivity to TKI. Among the cases reported, two cases were complex mutations of 19 del combined with V786M (15, 16). Compared with common mutations as 19 del or L858R, uncommon or complex mutations are prone to be less sensitive to EGFR TKIs (17).

EGFR TKI use after ICI has been reported with higher chances of interstitial pneumonitis (18). Therefore, osimertinib was not considered for fear of severe adverse events within 3 months of ICI exposure. Osimertinib was proposed to have an immunomodulatory function, which may increase the possibility of ICI-induced pneumonitis. Currently, there were no reported cases of another third-generation EGFR TKI almonertinib. However, the impact of ICI on sequential use of other third-generation TKIs still requires further investigation. Of note, first-line therapy resulted in a best response of stable disease for 7 months and no serious adverse events were observed. In oncogene-addicted NSCLC, ICIs are usually administered at the failure of other treatment options (19). Different subtypes of EGFR mutations might influence the efficacy of the PD-1 inhibitor in NSCLC (20).

Up to now, only eight cases with EGFR exon 20 V786M mutation have been reported in literature (**Table 1**). Among them, only one comprehensive case report was identified (7). Most of the patients with reported response evaluation responded to first-generation EGFR TKIs, except one patient with squamous carcinoma of the lung who experienced progressive disease.

**Table 1:** Cases reported of EGFR exon 20 V786M.

No.	Author	Year	Country	Mutation	Sex	Age	Histology	TKI	Treatment line	Best response
1	Current paper	2022	China	V786M	Female	69	Adenocarcinoma	**Gefitinib**	3rd	**PD**
								**Almonertinib**	4th	**PR**
2	Lu (21)	2019	China	V786M	Female	59	Adenocarcinoma	NR	NR	NR
3	Baek (17)	2015	Korea	V786M	NR	NR	NR	NR	NR	NR
4	Keam (22)	2014	Korea	V786M	Male	74	Adenocarcinoma	**Gefitinib**	1st	NR
5	Choi (23)	2013	Korea	V786M	NR	NR	NR	NR	NR	NR
6	Cho (24)	2012	Korea	V786M	Male	62	Squamous cell carcinoma	**Gefitinib**	1st	**PD**
7	Yi (16)	2009	Korea	19 del + V786M	Female	NR	Adenocarcinoma	NR	NR	NR
8	Kubota (15)	2008	Japan	19 del + V786M	Female	NR	Adenocarcinoma	**Erlotinib**	2nd	**PR**
9	Ludovini (7)	2008	Italy	V786M	Male	48	Adenocarcinoma	**Gefitinib**	1st	**CR**

NR, not reported; SD, stable disease; PD, progressive disease; PR, partial response; CR, complete response.

In summary, our data confirmed the oncogenic role of V786M mutation on EGFR in NSCLC. This case also highlights the role of the protein structure analysis of EGFR rare mutations as a useful predictor of TKI efficacy.

## Data Availability Statement

The datasets for this article are not publicly available due to concerns regarding participant/patient anonymity. Requests to access the datasets should be directed to the corresponding author.

## Ethics Statement

Ethical review and approval were not required for the study on human participants in accordance with the local legislation and institutional requirements. The patients/participants provided their written informed consent to participate in this study.

## Author Contributions

LP and QZ wrote the manuscript. LP, JS, and JL revised the manuscript and provided guidance. WL, SS, and JL performed the protein structure simulation. LP, MJ, XL, and QZ took care of the patient. All authors contributed to the article and approved the submitted version.

## Funding

This study was supported by a grant from the Medical Science Research Foundation of Health Bureau of Zhejiang Province (Grant number: 2022KY545) and a grant from the Administration of Traditional Chinese Medicine of Zhejiang Province (Grant number: 2022ZA021). The funding bodies played no role in the design of the study and collection, analysis, and interpretation of data and in writing the manuscript.

## Conflict of Interest

JS is the Editor-in-Chief of Oncogene and has sat on SABs for Vaccitech, Heat Biologics, Eli Lilly, Alveo Technologies, Pear Bio, Agenus, Equilibre Biopharmaceuticals, Graviton Bioscience Corporation, Celltrion, Volvox, Certis, Greenmantle, vTv Therapeutics, APIM Therapeutics, Bryologyx and Benevolent AI. He has also consulted with Lansdowne partners and Vitruvian. He chairs the Board of Directors for Xerion and previously BB Biotech Healthcare Trust PLC.

The remaining authors declare that the research was conducted in the absence of any commercial or financial relationships that could be construed as a potential conflict of interest.

## Publisher’s Note

All claims expressed in this article are solely those of the authors and do not necessarily represent those of their affiliated organizations, or those of the publisher, the editors and the reviewers. Any product that may be evaluated in this article, or claim that may be made by its manufacturer, is not guaranteed or endorsed by the publisher.

## Supplementary Material

Supplementary Figure 1 Second-line therapy of chemotherapy plus VEGFR-TKI.** (A–C)** Chest CT and brain MRI captured on April 21st, 2021. **(D–F)** Chest CT and brain MRI captured on June 3rd, 2021; **(G–I)** Chest CT and brain MRI captured on October 7th, 2021 (after second-line therapy). Significant abnormal findings noted (arrow). VEGFR, Vascular endothelial growth factor; TKI, Tyrosine kinase inhibitor.
