# Supplementary figures and images for: A Lung Cancer Patient Harboring a Rare Oncogenic EGFR Exon 20 V786M Mutation Responded to a Third-Generation Tyrosine Kinase Inhibitor: Case Report and Review of the Literature
Source: Front Oncol. 2022 May 18;12:912426. doi: 10.3389/fonc.2022.912426 (PMC9159765; doi:10.3389/fonc.2022.912426)

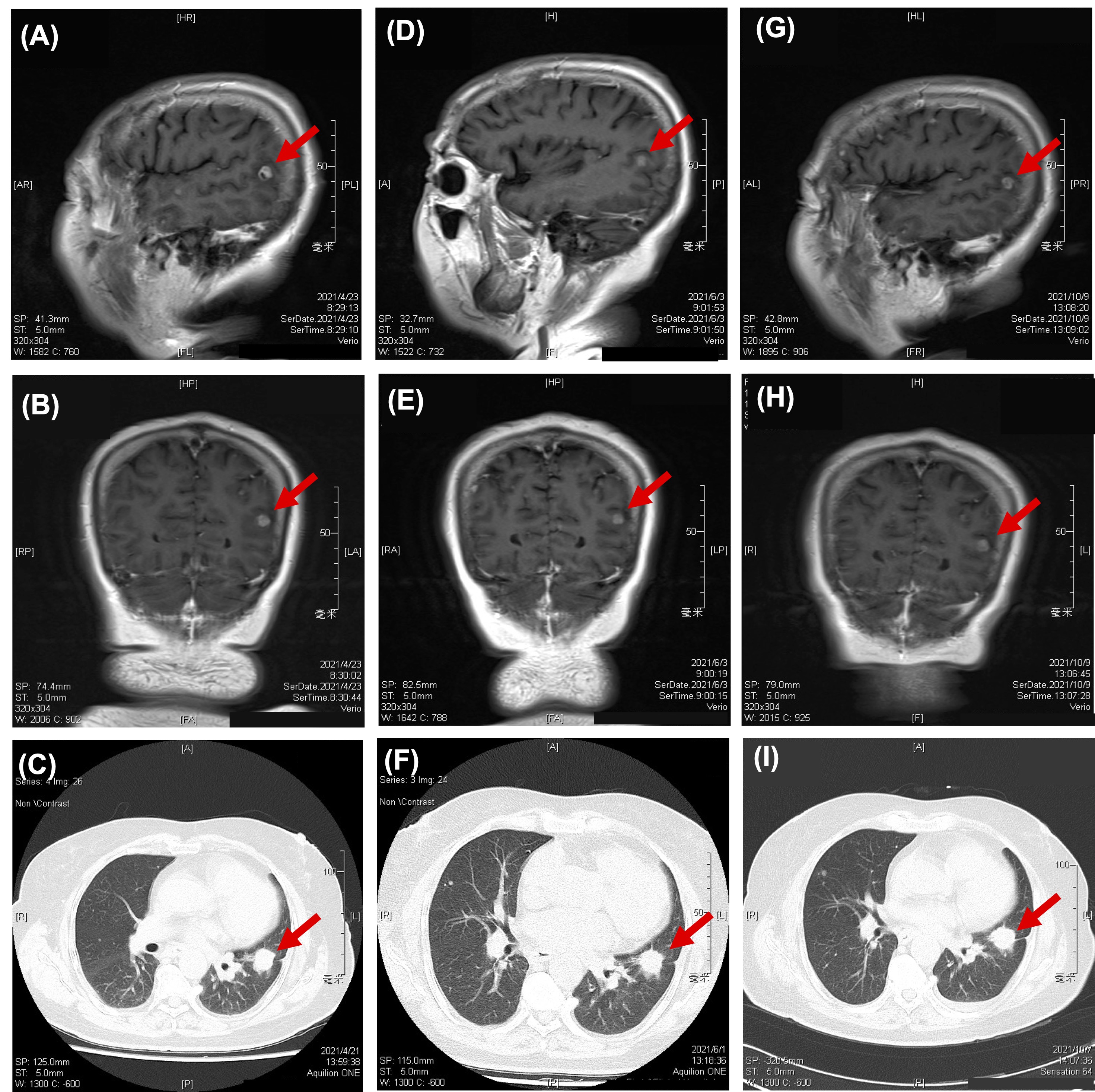

Supplement: Supplementary Figure 1 — Second-line therapy of chemotherapy plus VEGFR-TKI. (A–C) Chest CT and brain MRI captured on April 21st, 2021. (D–F) Chest CT and brain MRI captured on June 3rd, 2021; (G–I) Chest CT and brain MRI captured on October 7th, 2021 (after second-line therapy). Significant abnormal findings noted (arrow). VEGFR, Vascular endothelial growth factor; TKI, Tyrosine kinase inhibitor. [file Image_1.jpeg]
